# T-cell immunity against influenza virus does not require Th1 or Th17 master regulator transcription factors

**DOI:** 10.1016/j.mucimm.2025.08.005

**Published:** 2025-08-15

**Authors:** Kunal Dhume, Caroline M. Finn, Eugene Baffoe, Lauren A. Kimball, Siva N. Annamalai, Verónica Urdaneta-Páez, Jash Trivedi, Taj Azarian, Tara M. Strutt, K. Kai McKinstry

**Affiliations:** a Burnett School of Biomedical Sciences, Division of Immunity and Pathogenesis, College of Medicine, University of Central Florida, Orlando, FL, USA; b Burnett School of Biomedical Sciences, Division of Molecular Microbiology, College of Medicine, University of Central Florida, Orlando, FL, USA

**Keywords:** Influenza A virus, CD4 T cell, Th1, Th17, Heterosubtypic immunity, Superinfection

## Abstract

Transcriptional programming needed for CD4 T cell immunity against influenza A virus (IAV) is unclear. Most antiviral CD4 T cells fit Th1 criteria, but cells unable to develop Th1 identity, through deletion of the transcription factors T-bet and Eomesodermin, remain protective. These double knockout (DKO) cells produce Th17 cytokines and express the Th17 ‘master regulator’, Rorγt, supporting the concept that Th17 programming is needed for Th1-independent T cell immunity. Here, we directly tested requirements for Rorγt in promoting this mode of protection using T-bet/Eomesodermin/Rorγt triple knockout (TKO) mice. We show that Th17 functions are dramatically reduced in TKO cells but that they can nevertheless transfer protection against IAV to unprimed wildtype mice. Furthermore, TKO mice efficiently clear primary IAV infection, resist lethal bacterial superinfection, and generate antibody-dependent immunity against reinfection with the same virus. Finally, T cell-dependent heterosubtypic immunity is similarly effective in IAV-primed TKO, DKO, and wildtype mice. However, strikingly different T cell response patterns and inflammatory landscapes underlie these protective outcomes, highlighted in TKO mice by Th2-linked components not typically associated with efficient viral clearance. Our results reveal an unexpected degree of flexibility in T cell responses able to combat IAV, underscoring their potential to enhance vaccine strategies.

## Introduction

Current Influenza Virus (IAV) vaccines focus on generating neutralizing antibodies (Abs) against the surface glycoproteins hemagglutinin (HA) and neuraminidase (NA) expressed by IAV strains that are predicted to predominate during the upcoming ‘flu’ season. This approach requires annual vaccine reformulation due to the evolution of IAV’s surface antigens and is of limited efficacy when vaccine-primed Abs do not match targets on circulating IAVs. IAV-specific CD4 T cells have been shown to be protective in animal and clinical studies, even in the absence of preexisting neutralizing Abs.^1–5^ Furthermore, T cells recognize epitopes that are highly conserved across IAV strains. Such T cell responses underpin ‘heterosubtypic’ immunity, which can protect individuals primed by or vaccinated against one IAV strain against reinfection with IAVs expressing different HA/NA combinations.^6^ T cell immunity thus offers a compelling platform for ‘universal’ IAV vaccination able to protect against a broad array of IAVs.^7^ A prerequisite for optimizing such strategies is a clear understanding of the transcriptional regulation needed to promote effective T cell-dependent IAV clearance.

Most T cells primed by IAV and other viruses are marked by Th1 (CD4^+^) or Tc1 (CD8^+^) hallmarks typified by expression of the ‘master regulator’ transcription factor, T-bet, its paralog, Eomesodermin (Eomes), and production of the signature Th1 cytokine, IFNγ. While these and broader Th1 criteria are the focus of most clinical and experimental analysis, they do not provide reliable correlates of protection. For example, not only has T cell-mediated protection against IAV often been found not to require IFNγ,^8–11^ but IFNγ is emerging as a major driver of immunopathology.^10–15^ Furthermore, *Tbx21*^*−/−*^ (T-bet KO) and wildtype (WT) mice clear primary and heterosubtypic IAV infections with similar kinetics,^16–17^ indicating that canonical Th1 induction is not obligatory for effective T cell immunity. IAV-infected T-bet KO mice also survive infection with doses of *Streptococcus pneumoniae* (*Spn*) that rapidly kill IAV-infected WT mice.^17^ As bacterial superinfections are strongly linked with IAV-associated hospitalizations and deaths,^18^ a potential advantage of harnessing Th1-independent T cell responses against IAV is the amelioration of superinfection risk.

While less abundant than Th1 responders, IAV-specific IL-17-producing T cells, as well as Th17-associated cytokines, are increasingly linked with positive outcomes.^5,19–25^ To assess how Th17 functions impact IAV infection in the absence of concurrent Th1 responses, we recently studied IAV infection in T-bet KO mice also conditionally deficient in T cells for Eomes. These T-bet/Eomes double knockout (DKO) mice develop strong antiviral Tc17/Th17 responses, marked by expression of the Th17 ‘master regulator’, Rorγt, and production of the signature Th17 cytokine, IL-17A, without concomitant Th1/Tc1 functionality. We found that WT and DKO mice similarly clear primary and heterosubtypic IAV infections, and, using IAV-specific effector cells generated *in vitro*, that DKO Th17 cells and WT Th1 cells both similarly transfer protection to unprimed WT mice against otherwise lethal IAV challenge.^26^ These results imply that Rorγt-dependent Th17 programming is required for an alternative, Th1-independent mode of T cell protection.

Here, we investigated requirements for Rorγt expression by T cells in promoting the protective capacity of Th1-independent T cell responses against IAV. To do so, we crossed Rorγt-deficient mice with DKO mice to generate T-bet/Eomes/Rorγt triple knockout (TKO) mice. We show that Th17 functions are strongly curtailed in TKO CD4 T cells responding against IAV, while hallmarks of Th2 responses, that are tied to ineffective IAV control,^27^ are dramatically increased. Nevertheless, IAV-specific TKO cells primed in Th17 conditions are equally effective as DKO Th17 cells in transferring protection to unprimed WT mice. Furthermore, TKO mice clear primary IAV infection as effectively as DKO mice and resist *Spn* superinfection-induced morbidity. Finally, IAV-priming generates protective Ab-dependent homotypic immunity and T cell-dependent heterosubtypic immunity in TKO mice despite considerably altered T cell responses and lung inflammatory environments characterized by increased Th2 signatures. Our results thus reveal unexpected transcriptional flexibility underlying T cell-mediated protection against IAV and underscore the need to identify more precise correlates of protection beyond those tied to canonical T cell polarization states.

## Materials and methods

### Mice

All mice were obtained from The Jackson Laboratories on a C57BL/6J (B6) background and were housed and bred at the University of Central Florida’s Lake Nona Vivarium. T-bet/Eomes DKO mice were bred by crossing *Tbx21*^*−/−*^ mice (#004648), with *Eomes*^fl/fl^ (#017293) mice that had been crossed to CD4-*Cre* transgenic mice (#017336). T-bet/Eomes/RORγt (TKO) mice were generated by breeding DKO mice with *Rorc*^−/−^ mice (#007571). Some DKO and TKO mice expressed the OT-II TcR recognizing the chicken ovalbumin (OVA) peptide (aa 323–329). CD45.1^+^ B6 mice (#033076) were used as hosts for transfer experiments. Mice used for experiments were at least 8 weeks old. Male and female mice were used, with similar results found for both sexes. TKO mice displaying signs of thymoma were excluded from experiments. All protocols were approved by the University of Central Florida’s Institutional Animal Care and Use Committee (PROTO202300114).

### In vivo Ab treatment and infections

Stocks of PR8, PR8-OVA_II_, and A/Philippines were grown in allantoic cavities of embryonated chicken eggs and characterized at the Trudeau Institute. Mice were infected i.n. under isoflurane anesthesia with 50 μL of PBS containing the virus. Morbidity (ruffled fur, bent posture, absence of movement, and weight loss) was examined daily, and mice were euthanized if humane endpoints were reached.

In some experiments, 500 μg of neutralizing Abs against IL-6 (MP5–20F3) and TGFβ (1D11.16.8), or IL-4 (11B11) was administered i.p in 200 μl of PBS on 0,2,4 and 6 dpi with IAV. T cells were depleted by giving 300 μg of anti-CD4 (GK1.5) and/or anti-CD8 (2.43) Ab on days −3, −1, 1, and 3 dpi by i.p. injection. All of these Abs were sourced from BioXCell.

In some experiments, mice were challenged with a sublethal dose (10^3^ CFU) of mouse-passaged *Spn* strain D39^28^ i.n. in 50 μL of PBS under isoflurane anesthesia. *Spn* CFU were determined by homogenizing lungs in 1 ml of PBS and plating serial dilutions (10^−1^ to 10^−11^) on Trypticase^™^ Soy Agar (TSA II^™^) with 5 % Sheep Blood plates (BD Biosciences). Plates were incubated overnight at 37 °C with 5 % CO_2_.

### Naive CD4 T cell isolation, effector generation, and transfer

Naive OT-II cells were isolated from single cell suspensions of spleen and lymph nodes that were incubated on nylon wool for 1 hr (Polysciences) to enrich for T cells followed by Percoll (MilliporeSigma) gradient separation to isolate small, resting cells. CD4^+^ cells were then positively selected using CD4 microbeads (Miltenyi Biotec).

To generate effector cells, naive CD4 T cells were plated 1:1 with antigen presenting cells in RPMI 1640 media with 2 mM L-glutamine, 100 μg/mL penicillin/streptomycin (Invitrogen), 10 mM HEPES (Gibco), 50 μM 2-mercaptoethanol (MilliporeSigma), and 7.5 % FBS (Hyclone). Antigen presenting cells were prepared by incubating the CD90.2^−^ fraction of splenocytes from unprimed mice, obtained using CD90.2 microbeads (Miltenyi Biotec), with LPS (MilliporeSigma) and dextran sulphate (MilliporeSigma) for 2 days followed by irradiation prior to use. All cultures received OVA_II_ peptide and IL-2 at 11 ng/mL. Th17 cultures included anti-IFNγ Ab (XMG1.2) at 15 μg/mL, anti-IL-4 Ab (11B11) at 15 μg/mL, TNF at 10 ng/mL, TGFβ at 0.5 ng/mL, IL-21 at 50 ng/mL, IL-1b at 10 ng/mL, IL-23 at 25 ng/mL, and IL-6 at 20 ng/mL. Th1 cultures included IL-12 at 2 ng/mL and anti-IL-4 (15 μg/mL). Th2 cultures included IL-4 at 15 ng/mL and anti-IFNγ Ab (15 μg/mL). Abs were sourced from BioXcell, and cytokines from Peprotech. Mice received CD4 T cells under isoflurane anesthesia by *retro*-orbital (r. o.) transfer in 200 uL of PBS.

### Flow cytometry

Single cell suspensions were stained for 20-30 min on ice with fluorochrome-labeled Abs in FACS Buffer (PBS containing 0.5 % BSA and 0.02 % Sodium Azide) containing 1 μg of anti-FcR (2.4G2). The following Abs were used for surface staining of T cells: CD4 (RM4.5), CD8α (53-6.7), CD90.2 (53-2.1), CD44 (IM7), CD45.2 (104), NKG2A/C/E (20d5), CXCR5 (SPRCL5), PD-1 (J43), and CD25 (PC61). Innate immune populations were determined by gating as previously described^29^ using Zombie NIR fixable viability dye (Biolegend) prior to staining with MHC-II (M5/114.15.2), CD90.2 (53-21) CD45.2 (104), NK1.1 (PK136), CD49b (DX5), γδ TcR (eBioGL3), CD3 (17A2), Siglec F (E50-2440), CD11b (M1/70), Gr-1 (RB6-8C5). Samples were washed and fixed in 1 % paraformaldehyde prior to data acquisition.

Cells analyzed for intracellular cytokine production were stimulated for 4 hrs with 10 ng/mL PMA (MilliporeSigma) and 50 ng/mL ionomycin (MilliporeSigma). Brefeldin A (MilliporeSigma) was added after 2 hrs. The cells were then stained with surface markers, fixed in 4 % paraformaldehyde, permeabilized with saponin buffer (0.1 % saponin and 0.1 % NaN_3_ in PBS), and then stained with Abs against: IFNγ (XMG1.2), IL-2 (JES6-5H4), TNF (MP6-XT22), IL-17A (TC11-18H10.1), IL-17F (9D3.1C8), IL-22 (Poly5164), IL-4 (11B11), IL-5 (TRFK5), IL-13 (eBio13A), IL-10 (JES5-16E3), Granzyme B (GB11), and Perforin (S16009A).

Intranuclear staining was done using the FoxP3/Transcription factor staining buffer kit (ThermoFisher) as per manufacturer’s instructions using Abs against Rorγt (B2D), GATA-3 (TWAJ), Bcl-6 (BLC-DWN), FoxP3 (3G3), Ki-67 (SolA15), c-Maf (T54-853), and Blimp-1 (5E7).

To detect polyclonal IAV-specific T cells, cell suspensions were incubated with for 1 hr with fluorochrome-labeled IAV-specific MHC-I (NP_366–374_ and PA_224–233_) tetramers at room temperature, or with a MHC-II (NP_311–325_) tetramer at 37 °C prior to surface staining.

All FACS Abs were purchased from ThermoFisher, BioLegend, or BD Biosciences. The IAV-specific tetramers were supplied by the NIH tetramer facility. Cytoflex S (Beckam Coulter) or FACSCanto II (BD Biosciences) cytometers were used to acquire data. All analysis was done using FlowJo software (BD Biosciences).

### Real-time PCR

Viral load was quantified from whole-lung tissues homogenized in TRIzol (Thermo Fisher). RNA was purified by phase separation, RNeasy kit (Qiagen), and DNase treated (ThermoFisher). SuperScript II reverse transcriptase (Thermo Fisher) and random primers were used to reverse transcribe 2.5 μg of RNA into cDNA. The polymerase (PA) gene in 50 ng of cDNA was quantified using the following probes and primers: probe, 5′−6-FAM-CCAAGTCATGAAGGAGAGGG AATACCGCT-3′; reverse primer, 5′-CATTGGGTTCCTTCCATCCA-3′; and forward primer, 5′-CGGTCCAAATTCCTGCTGA-3′. A standard curve using a PA-containing plasmid with known concentration was used to calculate total PA copy number. Samples were run on a QuantStudio 7 Flex Real-Time PCR system (Applied Biosystems).

### RNASeq

Total RNA was obtained from effector CD4 T cells harvested into TRIzol (ThermoFisher). RNA was isolated by phase separation, purified and DNase treated as described above. Libraries were prepared using Illumina’s Stranded Total RNA Prep Ligation with Ribo-Zero Plus Kit and 10 bp unique dual indices (UDI). Paired end 150 bp reads were sequenced using a NovaSeq X Plus. Raw sequencing data were analyzed using a custom bioinformatics pipeline available at https://github.com/J22160/DualRNASeq_Pipeline. Differential gene expression analysis and KEGG pathway enrichment analysis was performed using RNAlytics available at https://github.com/J22160/RNAlytics. For identifying differentially expressed genes (DEGs), the significance threshold was set at |log_2_(fold change)| ≥ 2 and adjusted p-value < 0.05. The raw data files are deposited in the Gene Expression Omnibus, accession number GSE291839.

### Detection of Abs, cytokines, and chemokines

IAV-specific IgG was measured in convalescent serum from A/PR8-primed mice at 45 dpi by ELISA. ELISA plates (Nunc) were coated overnight at with PR8 at 4 °C then washed with PBS-Tween 20 with 1 % BSA and blocked with PBS with 2 % BSA. Serum samples were serially diluted in PBS with 2 % BSA and were incubated for 2 hrs at room temperature. After washing, HRP-conjugated Ab for total mouse IgG (1030–05; Southern Biotech) was added in PBS with 2 % BSA for 1 hr at room temperature. After washing, o-phenylenediamine dihydrochloride (MilliporeSigma) was added. The reaction was stopped with 25 % sulfuric acid and signal was measured at 492 nm. Endpoint titers were defined as the last serum dilution giving a reading above 2 SD of negative control values.

Luminex multiplexing kits (MilliporeSigma) were used to determine concentrations of chemokines and cytokines in homogenized lung tissues using a Luminex 200 reader (Bio-Rad). Heat maps for Luminex data were generated using Morpheus (https://software.broadinstitute.org/morpheus).

### Statistical analysis

Significant differences between two normally distributed groups were determined by comparing means using unpaired, two-tailed, Student *t* tests (α = 0.05). When variances were found to be different, Welch correction was applied. Multiple means were compared using One-way ANOVA analysis with Bonferroni correction. Significance is shown by ****p < 0.0001, ***p < 0.001, **p < 0.005 and *p < 0.05. SD is represented by error bars.

## Results

### Rorγt regulates Th17 functionality in vitro

**Fig. 1.**
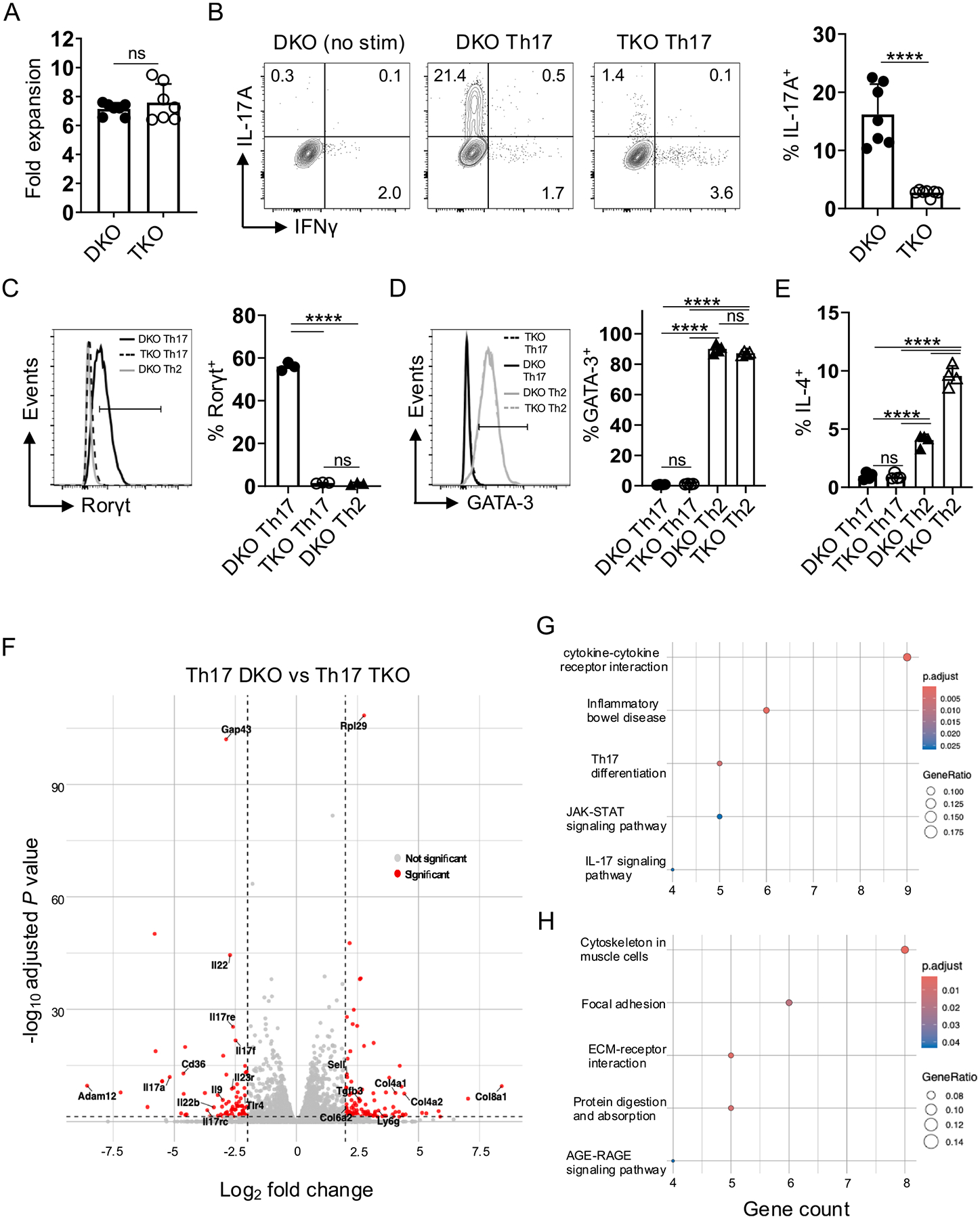
Rorγt regulates in vitro Th17 differentiation of DKO CD4 T cells. Naive DKO or TKO OT-II cells were plated in Th17 culture conditions with cognate peptide and irradiated antigen presenting cells. (A) Fold expansion of 7 individual cultures each for DKO and TKO cells. (B) Representative IL-17A and IFNγ production by DKO and TKO Th17 effectors after restimulation and the frequency of IL-17A^+^ cells from individual cultures. (C) Representative Rorγt expression by DKO and TKO Th17 cells, with control staining from DKO cells primed in Th2 conditions (left), and the frequency of Rorγt^+^ cells in 3 individual DKO and TKO cultures (right); 1 of 3 experiments. (D) Representative staining for GATA-3 (left) and expression by DKO and TKO Th17 and Th2 cells from individual cultures (right); 1 of 3 experiments. (E) The frequency of IL-4^+^ cells detected in individual DKO and TKO Th17 and Th2 cultures; 1 of 3 experiments. (F) Volcano plot depicting 191 differentially expressed genes (log_2_ fold change; *P* ≤ 0.05) between DKO and TKO Th17 cells by RNAseq analysis from 3 independent replicates per condition. Pathways enriched for differentially expressed genes (G) downregulated or (H) upregulated by TKO vs DKO effectors determined by KEGG analysis.

To study how Rorγt impacts antiviral CD4 T cell responses that lack transcription factors required for Th1 differentiation, we crossed *Rorγc*^−/−^ mice^30^ to *Tbx21*^−/−^ mice expressing CD4-driven *Cre* and a floxed allele of *Eomes* to generate T-bet/Eomes/Rorγt triple knockout (TKO) mice. We first compared Th17 development *in vitro* by priming TKO and Tbet/Eomes double knockout (DKO) CD4 T cells expressing the OT-II T cell receptor (TcR) in well-established Th17 conditions. While TKO and DKO effector expansion was similar at 4 days of culture (Fig. 1A), the frequency of IL-17A^+^ cells detected after restimulation was decreased ~ 90 % in TKO vs DKO cultures (Fig. 1B), which aligns with the absence of Rorγt expression in TKO cells (Fig. 1C). We also assessed effectors primed in Th2 conditions. Rorγt was not induced in DKO cells in Th2 conditions (Fig. 1C), while > 80 % of DKO and TKO effectors similarly expressed the Th2 ‘master regulator’, GATA-3,^31^ which was not upregulated in Th17 conditions (Fig. 1D). Furthermore, while <1 % of Th17-primed DKO or TKO effectors produced IL-4, the signature Th2 cytokine, IL-4^+^ cells were readily detected in DKO Th2 cultures and were increased 2-fold in TKO Th2 cultures (Fig. 1E). Thus, while TKO cells have a heightened capacity for IL-4 production vs DKO cells when stimulated under Th2 conditions, neither DKO or TKO cells display Th2 attributes in Th17-polarizing conditions.

To determine Rorγt’s impacts more broadly, DKO and TKO cells cultured in Th17 conditions were analyzed by RNAseq. We identified 191 differentially expressed genes (Fig. 1F), including reduced expression in TKO cells of many Th17 hallmarks like *IL17a*, *Il17f*, *Il17rc*, *Il17re*, and *IL22* (Supplemental Table 1). In line with this, KEGG analysis revealed downregulated genes in pathways including cytokine-cytokine receptor interaction, IL-17 signaling pathway, Th17 cell differentiation, and JAK-STAT signaling (Fig. 1G). Genes upregulated in TKO cells were enriched in pathways including ECM-receptor interaction, protein digestion and absorption, focal adhesion, and cytoskeleton in muscle cells (Fig. 1H). This analysis reveals a wide breadth of Rorγt-dependent regulation that could impact Th17-prone DKO CD4 T cell responses primed *in vivo* by IAV infection.

### Rorγt regulates Th17 functionality during IAV infection

**Fig. 2.**
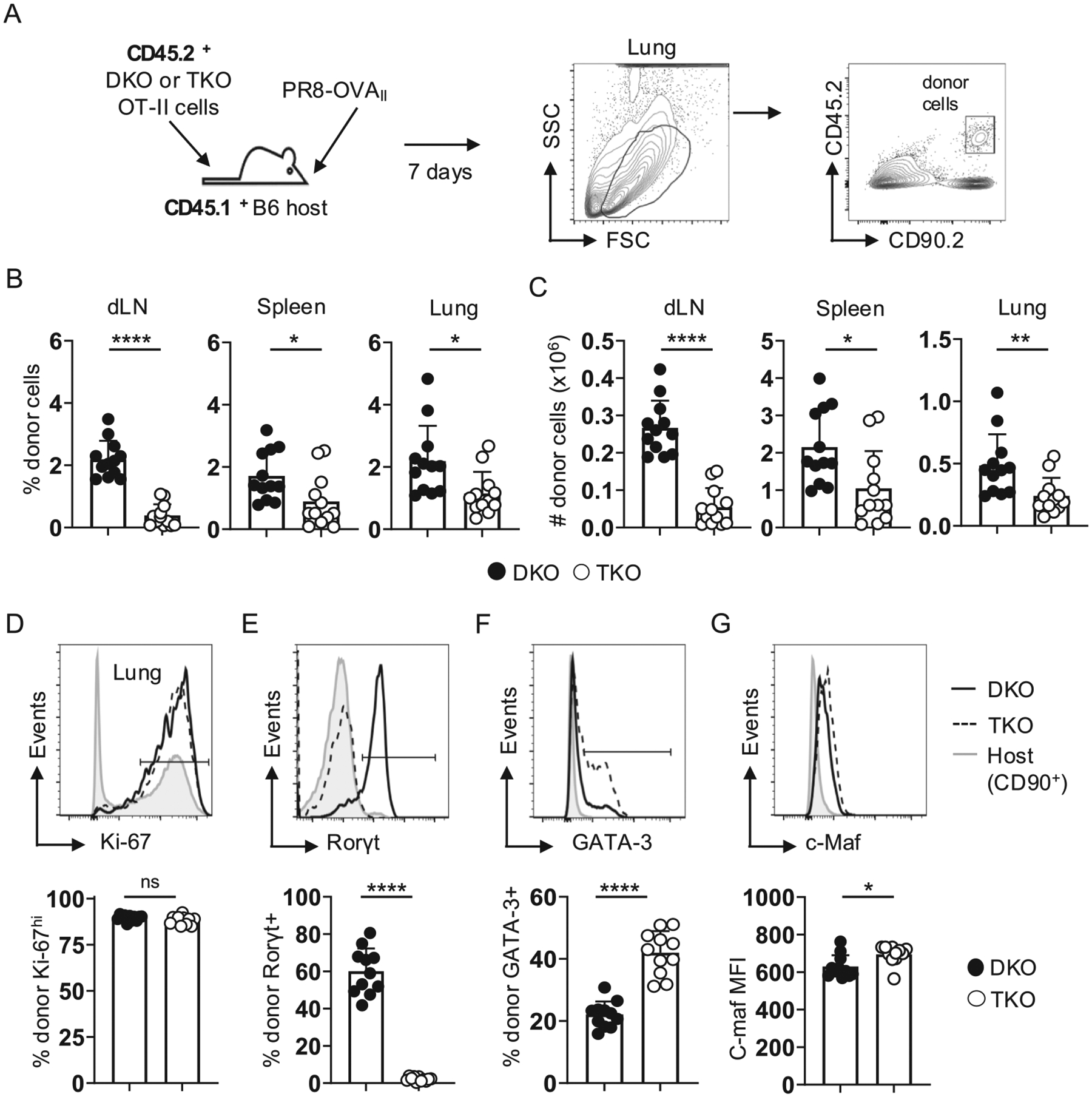
Rorγt maximizes expansion and restricts Th2 transcription factor expression in DKO CD4 T cells responding to IAV. (A) 5×10^5^ naive CD45.2^+^ DKO or TKO OT-II cells were transferred to unprimed CD45.1^+^ WT hosts that were then challenged with 0.25 LD_50_ PR8-OVA_II_, with representative gating to identify donor cells at 7 dpi shown. (B) the frequency and (C) number of donor cells detected in stated organs at 7 dpi; 12 mice/group pooled from 3 experiments. (D) Representative donor cell Ki-67 expression from the lung with control staining from total host T cells as a shaded histogram (top) and summary analysis (bottom) from 12 mice/group pooled from 3 experiments. Similar representative staining and summary analysis for (E) Rorγt, (F) GATA-3, and (G) c-Maf from DKO and TKO cells from the same mice.

We next transferred 5×10^5^ naive CD45.2^+^ DKO or TKO OT-II cells to unprimed WT CD45.1^+^ B6 mice and then challenged them with a sublethal dose of the IAV strain PR8-OVA_II_, which is recognized by the OT-II TcR, and analyzed the CD45.2^+^ donor cells at 7 dpi, the peak of the DKO OT-II response^26^ (Fig. 2A). In this model, infection induces a WT inflammatory environment in which the donor cells respond. Across several experiments, the frequency and number of TKO cells reached only about one-third of DKO cells in the draining mediastinal lymph node (dLN), and about one-half in the spleen and lungs (Fig. 2 B and C). Expression of the proliferation marker, Ki-67, by DKO and TKO cells in secondary lymphoid organs (Supplemental Fig. 1), and lungs (Fig. 2D) was, however, similar. Thus, in contrast to effector development *in vitro*, Rorγt is required for optimal expansion of DKO cells primed by IAV infection.

About 60 % of DKO cells in the lungs were Rorγt^+^ (Fig. 2E), while very few Rorγt^+^ DKO cells were detected in the spleen or dLN (Supplemental Fig. 1), consistent with our previous analysis.^26^ In contrast, about twice as many TKO cells in the lungs expressed GATA-3 (Fig. 2F), with much reduced and similar GATA-3 expression by DKO or TKO cells in the dLN and spleen (Supplemental Fig. 1). TKO cells in the lungs also expressed slightly higher levels of c-Maf (Fig. 2G), a transcription factor tied to Th2, but also to Th17 responses in some settings,^32–34^ while expression of c-Maf by both donor populations in the dLN and spleen was similar and lower than in the lungs (Supplemental Fig. 1).

### Reduced but not absent Th17 functionality in TKO effectors

**Fig. 3.**
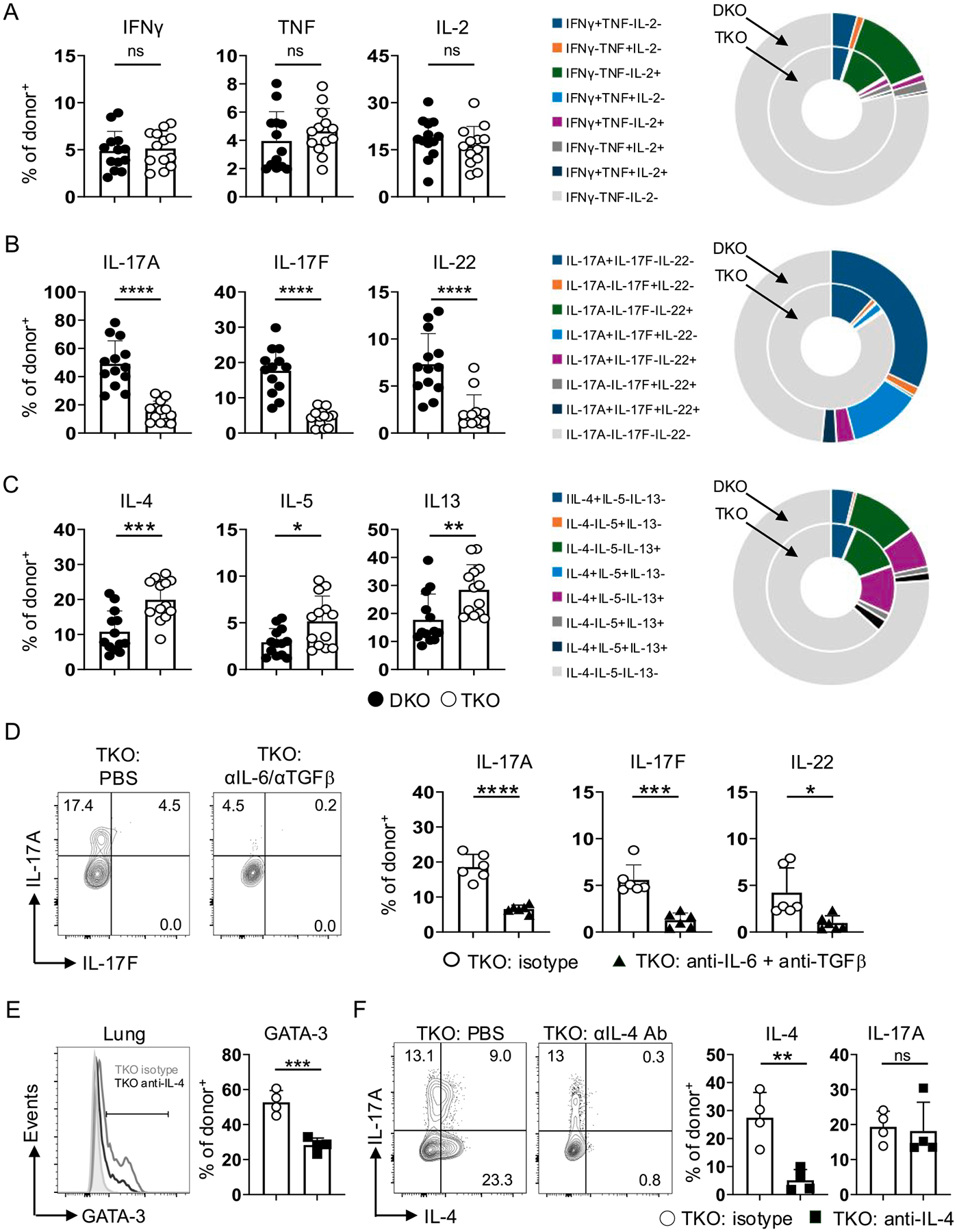
Loss of Th17 and gain of Th2 function by TKO vs DKO CD4 T cells responding against IAV. 5×10^5^ naive CD45.2^+^ DKO or TKO OT-II cells were transferred to unprimed CD45.1^+^ WT hosts that were then challenged with 0.25 LD_50_ PR8-OVA_II_. DKO and TKO cells in the lungs of individual mice were assessed for cytokine expression after restimulation at 7 dpi. (A) IFNγ, TNF, and IL-2 production (left) with summary analysis of Th1 cytokine production patterns (right). (B) IL-17A, IL-17F, and IL-22 production (left) with summary analysis of Th17 cytokine production patterns (right). (C) IL-4, IL-5, and IL-13 production (left) with summary analysis of Th2 cytokine production patterns (right). 13 mice per group pooled from 4 experiments for A-C. WT mice receiving TKO donor cells were treated with IL-6 and TGFβ neutralizing Abs or PBS alone during PR8-OVA_II_ infection. (D) Representative staining for IL-17A and IL-17F from control and Ab treated mice (left) and summary analysis of IL-17A, IL-17F, and IL-22 from 6 mice/group pooled from 2 experiments. WT recipients of TKO OT-II cells were treated with IL-4 neutralizing Abs and shown is (E) representative GATA-3 expression (WT host CD90.2^+^ cells as shaded histogram) and summary analysis from individual mice, and (F) representative IL-4 and IL-17A production and summary analysis from TKO cells =4 mice per group (right), 1 of 2 experiments.

We next assessed cytokine production capacity of the IAV-primed TKO and DKO OT-II cells in the lungs using the transfer model described above (for representative cytokine staining see Supplemental Fig. 2). Few TKO or DKO effectors produced the Th1-linked cytokines IFNγ and TNF, and production of IL-2 was also similar (Fig. 3A). As expected from *in vitro* analysis, frequencies of TKO cells producing the Th17 cytokines IL-17A, IL-17F, and IL-22, were reduced more than two-fold vs DKO cells (Fig. 3B) but nevertheless were clearly detectable compared to their near absence during WT OT-II responses.^26^ In contrast, and in line with increased GATA-3 levels, frequencies of TKO effectors expressing the Th2-associated cytokines IL-4, IL-5, and IL-13, were nearly doubled vs DKO effectors (Fig. 3C).

IL-6 and TGFβ are required for DKO cells to gain Th17 identity during IAV infection.^26^ We next asked whether these signals are likewise needed for Th17 cytokine production by TKO cells. Treating IAV-infected host mice with IL-6 and TGFβ blocking Abs dramatically reduced IL-17A, IL-17F, and IL-22 production (Fig. 3D). On the other hand, treating mice with Abs to neutralize IL-4, the prime driver of Th2 differentiation, reduced the frequency of GATA-3^+^ TKO cells by ~ 2-fold (Fig. 3E) and IL-4^+^ cells by ~ 4-fold without impacting IL-17A production (Fig. 3F). Canonical Th-polarizing signals induced by IAV infection are thus required to promote Th17 and Th2 cytokine production by TKO CD4 T cells.

**Fig. 4.**
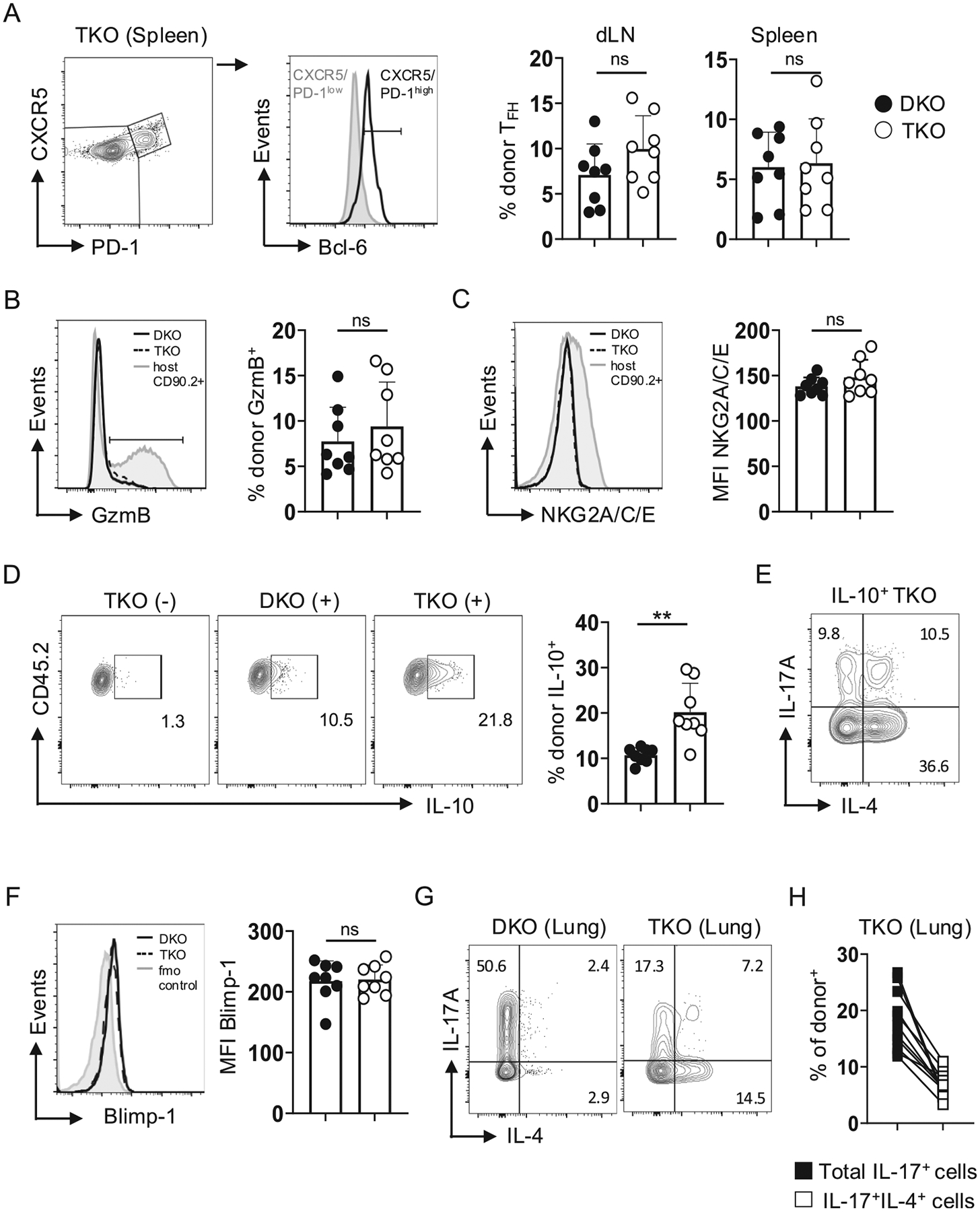
Rorγt is not needed for development of specialized CD4 T cell effectors. 5×10^5^ naive CD45.2^+^ DKO or TKO OT-II cells were transferred to unprimed CD45.1^+^ WT hosts that were then challenged with 0.25 LD_50_ PR8-OVA_II_. (A) Representative staining used to identify T_FH_ based on co-expression of CXCR5, PD-1, and Bcl-6 (left) with frequencies of DKO and TKO T_FH_ detected in the dLN and spleen (right) from individual mice. Representative staining and summary analysis for (B) Granzyme B (GzmB) and (C) NKG2A/C/E from donor cells responding in the lungs of individual mice. (D) Representative staining for IL-10 without (−) or with restimulation (+) and summary analysis from lung donor cells in individual mice. (E) Representative staining of IL-10^+^ TKO cells co-producing IL-17A, IL-4, or IL-17A and IL-4. (F) Representative Blimp-1 staining by lung DKO and TKO donor cells, with fluorescence minus one (fmo) control, and summary analysis from individual mice (8 mice/group pooled from 2 experiments for data shown in A, B, C, D, and F). (G) Representative IL-17A and IL-4 production by DKO and TKO cells in the lungs with (H) paired analysis of the same TKO OT-II cells responding in WT hosts comparing frequencies of total IL-17A^+^ cells to IL-17A^+^IL-4^+^ cells (11 mice pooled from 3 experiments).

We also used the OT-II transfer model to assess development of specialized effector subsets, not tied strictly to Th1, Th2, or Th17 programming, that can impact outcomes of IAV infection. The frequency of CXCR5^high^PD-1^high^Blc6^+^ follicular helper cells (T_FH_), which are largely restricted to secondary lymphoid organs during primary IAV infection,^35–36^ was similar between TKO and DKO OT-II cells in the dLN and spleen (Fig. 4A). Granzyme B^+^NKG2A/C/E^+^ cytotoxic CD4 T cells, on the other hand, are largely restricted to the lung during IAV infection,^37^ and expression of these molecules by DKO and TKO effectors in the lung was also similar (Fig. 4B and C). We previously found that although DKO OT-II express reduced Granzyme B and NKG2A/C/E vs WT cells, their cytotoxic functions are similar.^26^ Rorγt expression in DKO cells thus does not impact development of T_FH_ in secondary lymphoid organs, or cytotoxic CD4 T cells in the lungs during IAV infection.

Treg (FoxP3^+^CD25^+^) frequencies were very low within DKO and TKO OT-II cells in all organs tested (Supplemental Fig. 3). FoxP3^−^IL-10^+^ CD4 T cells develop in the lungs of IAV-infected mice and can impact outcomes, including repression of Th17 responses.^23^ Strikingly, the frequency of IL-10^+^ cells was nearly doubled in TKO vs DKO OT-II effectors (Fig. 4D). Interestingly, ~20 % of IL-10^+^ TKO cells co-expressed IL-17A, while ~ 45 % co-expressed IL-4, and ~ 10 % co-expressed IL-17A and IL-4 (Fig. 4E). Increased IL-10 production by Rorγt-deficient CD4 T cells has been seen in a colitis model in which reduced Blimp-1 expression contributed to this phenotype,^38^ but we found similar low levels of Blimp-1 expression by DKO and TKO cells at this timepoint (Fig. 4F). This is noteworthy too as Blimp-1 is a required driver of IAV-specific cytotoxic CD4 T cell development.^37^ Finally, given the unexpected level of co-production of IL-4 and IL-17 within the IL-10^+^ subset of TKO cells, we assessed IL-4 and IL-17 co-production by all TKO responders in the lung. While very few DKO IL-17A^+^ cells co-produced IL-4 (~5%), matching our previous findings,^26^ ~ 10 % of total TKO effectors co-produced IL-4 and IL-17A, representing about 30 % of all IL-17A^+^ cells across experiments (Fig. 4G and H). Together, these results highlight unexpected heterogeneity within Th-subset associated cytokine production patterns by TKO effectors primed by IAV.

### Th1 and Th17 but not Th2 TKO effectors transfer protection against lethal IAV infection

**Fig. 5.**
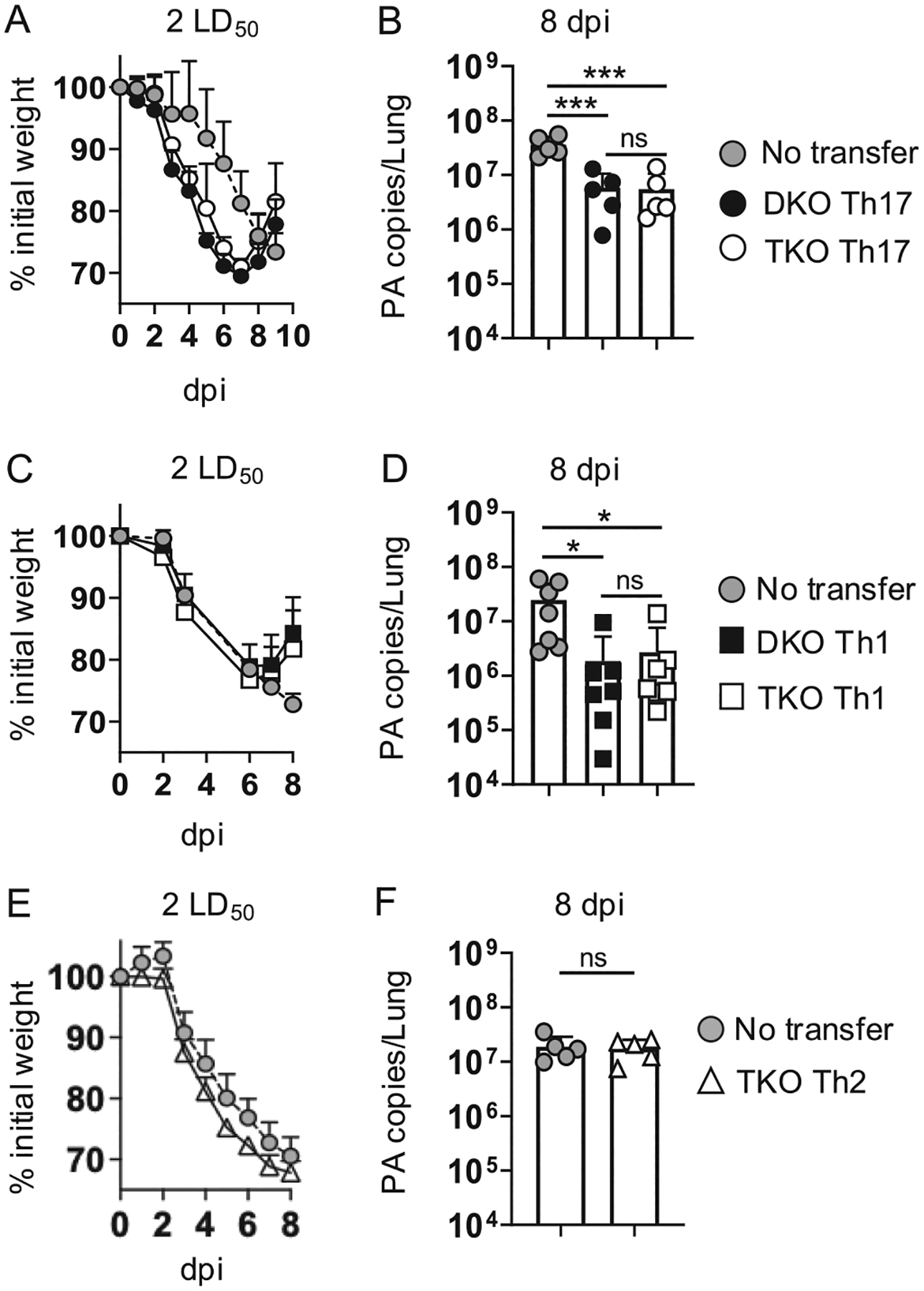
Th1 and Th17 but not Th2 primed TKO OT-II effectors transfer protection against IAV to unprimed mice. Naive DKO or TKO OT-II cells were primed in Th17 conditions as in Fig. 1. 3×10^6^ resulting effectors were transferred to unprimed WT mice that were then challenged with a lethal (2 LD_50_) dose of PR8-OVA_II_. (A) Weight loss and recovery of mice receiving DKO or TKO effectors and control mice not receiving cells; 4 mice/group; 1 of 2 experiments and (B) viral titer analysis from individual mice at 8 dpi; 5/group, 1 of 2 experiments. (C) Weight loss and recovery from mice receiving no cells or Th1-primed DKO or TKO effectors; 4 mice/group; 1 of 2 experiments, and (D) viral titer analysis at 8 dpi; 7 mice, pooled from 2 experiments. (E) Weight loss and (F) viral titer analysis at 8 dpi from mice receiving no cell transfer or 3×10^6^ TKO effectors primed in Th2 conditions; 5 mice/group, 1 of 2 experiments.

We previously showed that giving 3×10^6^ Th17-primed DKO OT-II effectors to unprimed WT mice can protect the mice against lethal PR8-OVA_II_ infection.^26^ To assess the extent to which Rorγt-dependent programming is required for this outcome, we gave 3×10^6^ DKO or TKO effectors primed in Th17 conditions as in Fig. 1 to unprimed B6 mice and then challenged them with a 2 LD_50_ dose of PR8-OVA_II_. While recipients of DKO and TKO effectors lost weight more quickly than did control mice, the transferred effectors similarly reversed weight loss vs mice not receiving cells that did not recover by 9 dpi (Fig. 5A). Furthermore, viral control mediated by DKO and TKO effectors as measured at 8 dpi was similar vs ~ 10-fold higher levels in control mice (Fig. 5B). Rorγt-dependent programming is thus not required during priming in Th17-polarizing conditions to generate CD4 T cell effectors that can effectively combat IAV.

DKO effectors primed in Th1 conditions are also protective, but they upregulate Rorγt during the IAV response *in vivo*,^26^ which could contribute, in part or in whole, to their antiviral impact. To test this, we gave unprimed mice 3×10^6^ Th1-primed DKO or TKO effectors. Weight loss patterns were similar between mice receiving Th1 effectors or not, but both DKO and TKO effectors similarly reversed weight loss (Fig. 5C) and controlled IAV levels (Fig. 5D) compared to mice not receiving cell transfer. Importantly though, TKO effectors primed in Th2 conditions were not protective as assessed by these measures (Fig. 5E and F), mirroring the failure of Th2-primed DKO effectors to protect unprimed mice against lethal PR8-OVA_II_ challenge in our previous studies.^26^ Thus, signals associated with Th17 or Th1, but not Th2 priming, are required to program TKO cells with the capacity to combat IAV.

### TKO mice resist Spn superinfection and generate homotypic and heterosubtypic immunity

**Fig. 6.**
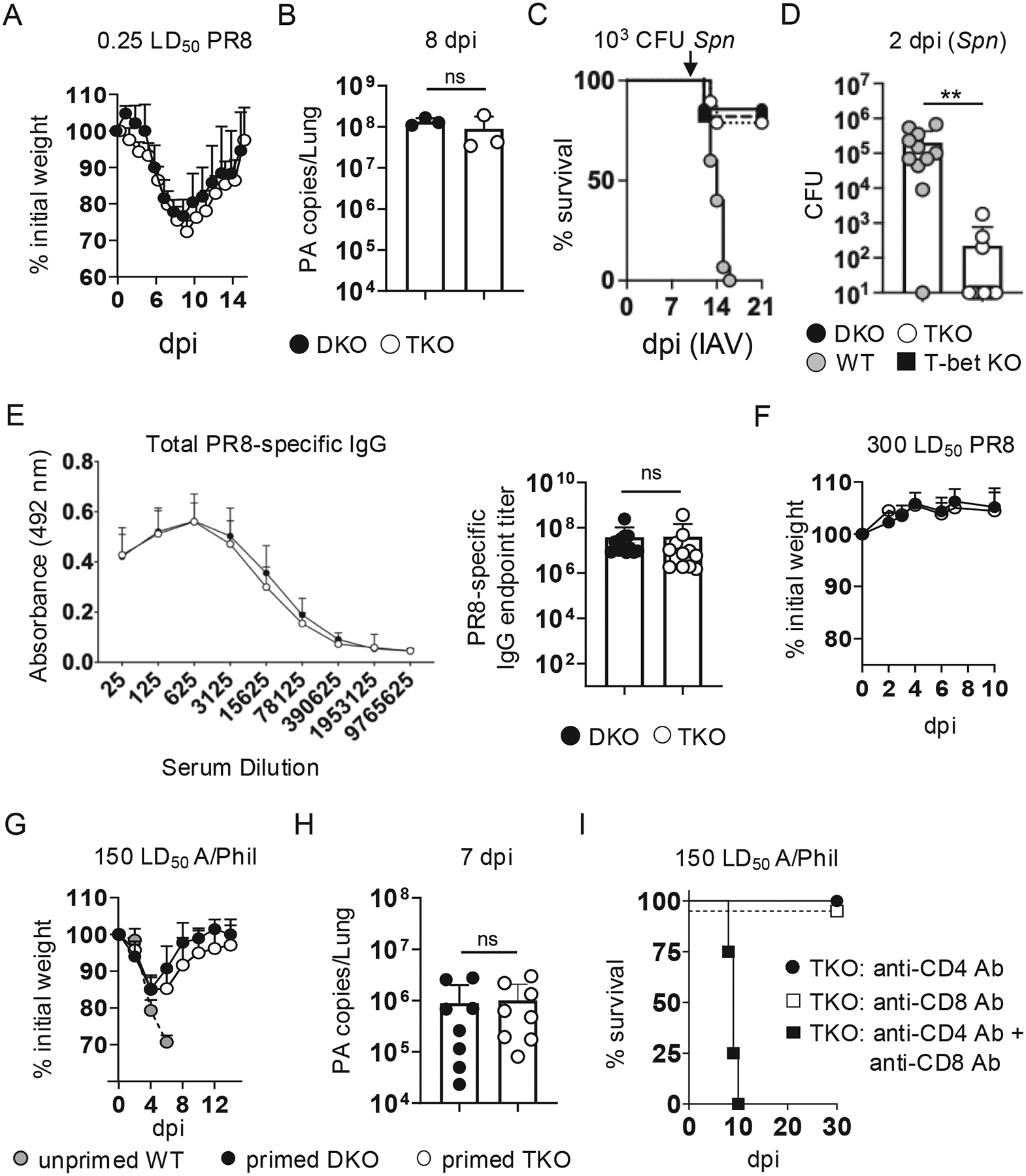
TKO mice clear primary IAV infection, resist bacterial superinfection, and generate protective homotypic and heterosubtypic immunity. Unprimed DKO or TKO mice were challenged with 0.25 LD_50_ PR8. (A) Weight loss and survival from 8 mice/group; 1 of 3 experiments. (B) Lung viral titers at 8 dpi; 3 mice/group, 1 of 2 experiments. (C) T-bet KO, WT, DKO, and TKO mice were challenged i.n. with 10^3^ CFU of *Spn* (D39) at 10 dpi with 0.25 LD_50_ PR8 (denoted by the arrow), with survival shown for 15 WT and TKO mice/group pooled from 3 experiments, 5 T-bet KO mice, and 8 DKO mice pooled from 2 experiments. (D) *Spn* CFU recovered from lungs of IAV-infected WT or TKO mice 2 days after D39 challenge; 11 mice/group pooled from 2 experiments. (E) Serum was collected from DKO and TKO mice 45 dpi with 0.25 LD_50_ PR8 and total PR8-specific IgG was determined by ELISA with absorbance vs dilution factor (left) and endpoint titer (right) for 12 mice/group pooled from 2 experiments. (F) DKO and TKO primed with PR8 as in (A) were rechallenged at 60 dpi with 300 LD_50_ PR8 with weight loss shown; 4 mice/group; 1 of 2 experiments. DKO and TKO mice were primed as in (A) and at 45 dpi were challenged with 150 LD_50_ A/Philippines (H3N2). (G) Weight loss from 5 mice/group and 3 unprimed WT mice; 1of 3 experiments. (H) Viral titers from primed mice at 7 dpi with A/Philippines; 8 mice/group pooled from 2 experiments. (I) PR8-primed TKO mice were treated with CD4 or CD8-depeleting Ab, or with both, prior to A/Philippines challenge, with survival shown for 4 mice/group; 1 of 2 experiments.

We next assessed outcomes after infecting DKO and TKO mice on a polyclonal T cell background with a sublethal, but pathogenic, 0.25 LD_50_ dose of PR8. *Rorc*^*−/−*^ mice fail to develop lymph nodes, but their formation is restored to an extent in mice lacking both Rorγt and T-bet.^39^ We found the presence of lymph nodes in TKO mice to be variable, with dLNs not detected in most infected TKO mice (Dhume and McKinstry, unpublished). Nevertheless, DKO and TKO mice both survived PR8 infection with similar weight loss patterns (Fig. 6A), and similar viral burden at 8 dpi (Fig. 6B). We previously showed that resolution of primary PR8 infection by DKO and WT mice is similar as determined by these parameters,^26^ and also found similar patterns in direct comparisons of WT and TKO mice (Supplemental Fig. 4). These results demonstrate comparable ability to clear primary IAV infection by WT, DKO, and TKO mice.

We next tested if IAV-infected TKO mice are susceptible to *Spn* superinfection-induced morbidity as resistance has been linked in several settings to the ability to mount rapid Th17-linked innate inflammatory responses against bacterial pathogens.^40–42^ To do so, we primed TKO mice with 0.25 LD_50_ PR8 and at 10 dpi, when the mice began to recover, challenged them i.n. with a sublethal (10^3^ CFU) dose of mouse-passaged *Spn*. We also assessed control groups of PR8-primed WT and T-bet KO mice, known to be susceptible and resistant to *Spn* superinfection, respectively,^17^ as well as DKO mice. While all WT mice succumbed within 5 days, ~ 80 % of T-bet KO, DKO, and TKO mice survived (Fig. 6C), indicating that resistance to *Spn* superinfection reported in T-bet KO mice does not require Rorγt-dependent regulation. Further supporting this conclusion, bacterial CFU were undetectable in the lungs of 8 out of 11 TKO mice analyzed 2 days after *Spn* challenge, with only 100 to 1000 CFU detected in the remaining 3 TKO mice assayed. In contrast, 10^4^ to 10^6^ CFU were detected in all but 1 of 11 WT mice assayed prior to superinfection-induced death (Fig. 6D).

We next asked if humoral immunity primed by IAV infection is impacted in TKO mice, as IL-17 signals have been tied to optimal B cell responses against IAV.^25,43^ Total IAV-specific IgG detected in the serum of PR8-primed TKO and DKO mice at 45 dpi was similar (Fig. 6E). To test the effectiveness of Ab-mediated immunity, DKO and TKO mice primed with 0.25 LD_50_ PR8 were rechallenged at 60 dpi with a supralethal (300 LD_50_) dose of PR8. All primed mice survived, with no signs of illness or weight loss (Fig. 6F), indicating robust, long-lived homotypic immunity.

We showed previously that memory CD4 or CD8 T cells can protect WT and DKO mice primed with PR8 (H1N1) against supralethal challenge doses of the heterosubtypic IAV strain A/Philippines (H3N2).^26^ The CD4-driven *Cre* used to generate the DKO and TKO mice used here deletes loxP-flanked gene expression by the double positive stage of thymic development,^44^ resulting in both CD4 and CD8 T cells lacking Eomes (in addition to T-bet, or T-bet and Rorγt). To determine if Rorγt expression is required for heterosubtypic immunity in DKO mice, we primed DKO and TKO mice with 0.25 LD_50_ PR8 and at 45 dpi challenged them with 150 LD_50_ A/Philippines. All PR8-primed mice survived, with similar patterns of weight loss and recovery, while unprimed WT mice succumbed within 9 dpi (Fig. 6G), with similar viral loads detected at 8 dpi in primed DKO and TKO mice (Fig. 6H). Heterosubtypic immunity in TKO mice was T cell-dependent, as depletion of CD4 and CD8 T cells, but neither subset alone, prior to A/Philippines challenge abolished protection (Fig. 6I), matching our previous findings with DKO mice.^26^ Rorγt-dependent programming is thus not required to establish or to mediate protective heterosubtypic CD4 or CD8 T cell responses in IAV-primed DKO mice.

### Hallmarks of T cell-dependent IAV immunity are strikingly altered in TKO vs DKO and WT mice

**Fig. 7.**
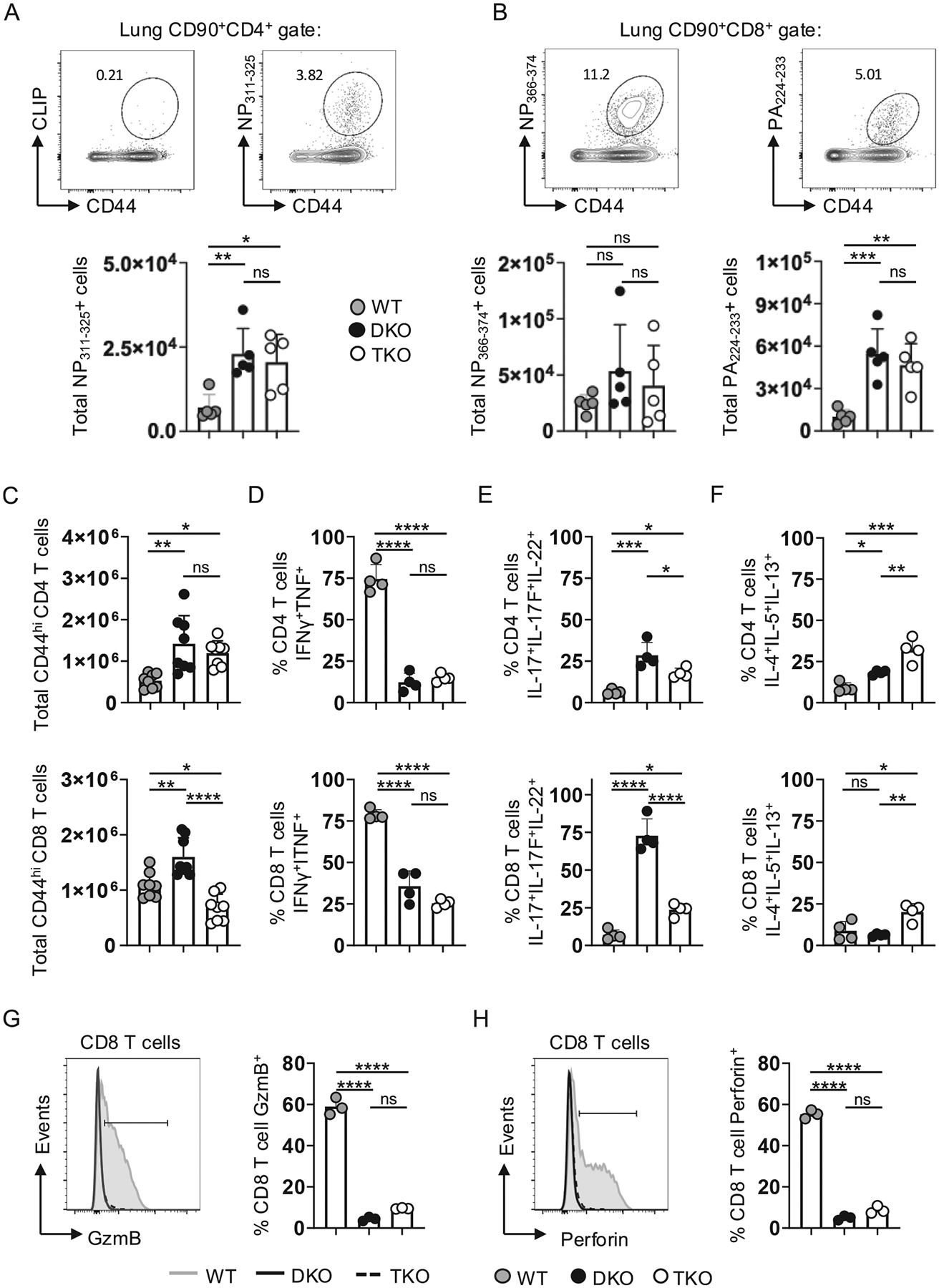
Distinct protective memory T cell signatures in WT, DKO, and TKO mice during heterosubtypic infection. WT, DKO and TKO mice were primed with 0.25 LD_50_ PR8, challenged at 45 dpi with 150 LD_50_ A/Philippines (H3N2), and analyzed at 4 dpi. (A) Representative staining of lung CD44^high^ CD4 T cells with the NP_311–325_ tetramer (top) with total ${\text{NP}}_{311-325}^{+}$ cells from individual mice (below) and (B) representative staining of CD44^high^ CD8 T cells with NP_366–374_ (left) and PA_224–233_ (right) tetramers with total tetramer^+^ cells below (5 mice/group; 1 of 2 experiments). (C) Total CD44^high^ CD4 (upper) and CD8 (lower) T cells in the lungs of PR8-primed WT, DKO, and TKO mice at 4 dpi; 8–9 mice/group; pooled from 2 experiments. CD44^high^ T cells in the lungs of PR8-primed mice were restimulated at 4 dpi with A/Philippines to detect total individual CD4 (upper) and CD8 (lower) T cells producing (D) Th1 (IFNγ and TNF) (E) Th17 (IL-17A, IL-17F, and IL-22) and (F) Th2 (IL-4, IL-5, IL-13) cytokines for 4 mice/group; 1 of 2 experiments. (G) Granzyme B (GzmB) and (H) Perforin expression by lung CD8 T cells for 3 mice/group; 1 of 2 experiments.

We next assessed recall responses by virus-specific memory T cells in the lungs of protected PR8-primed WT, DKO, and TKO mice at 4 dpi with A/Philippines, a timepoint preceding newly activated T cells reaching the lungs. We first quantified IAV-specific CD4 T cells using an MHC-II tetramer for the major I-A^b^ restricted nucleoprotein epitope NP_311–325_. The number of tetramer^+^ CD44^high^ CD4 T cells in the lungs was ~ 2-fold higher in DKO and TKO vs WT mice (Fig. 7A; see Supplemental Fig. 5 for gating used to identify CD44^high^ CD4 and CD8 T cells). We used two different tetramers to enumerate IAV-specific CD8 T cells: the NP_366–374_ epitope and the PA_224–233_ epitope. While the number of ${\text{NP}}_{366-374}^{+}$ cells was similar between strains, ~5-fold more ${\text{PA}}_{224-233}^{+}$ cells were seen in the lungs of DKO and TKO vs WT mice (Fig. 7B). These results suggest that T-bet and/or Eomes may have a significant role in shaping the epitope hierarchy within the IAV-primed CD8^+^ memory T cell pool, a possibility supported by studies in an LCMV model.^45^

To capture the anti-viral T cell response more broadly, we enumerated total memory (CD44^high^) CD4 and CD8 T cells in the lungs of PR8-primed mice at 4 dpi with A/Philippines as in our previous studies.^26^ Matching patterns seen with the NP_311–325_ tetramer, the number of CD44^high^ CD4 T cells was also increased ~ 2-fold in DKO and TKO vs WT mice (Fig. 7C, **upper panel**). CD44^high^ CD8 T cells, on the other hand, were highest in DKO lungs and were reduced ~ 30 % in WT and ~ 50 % in TKO lungs (Fig. 7C, **lower panel**). These patterns do not match those seen assessing the NP_366–374_ or PA_224–233_ responses in Fig. 7B. This may indicate broader shifts in Ag responsiveness, beyond TcRs targeting immunodominant epitopes, in the IAV-primed memory CD8 T cell compartment of DKO and TKO mice compared to the WT condition. To compare the relative cytokine production profiles of responding memory T cells across mouse strains we measured the sum frequencies of CD44^high^ CD4 and CD8 T cells producing individual Th1 (IFNγ and TNF), Th17 (IL-17A, IL-17F, and IL-22), or Th2 (IL-4, IL-5, and IL-13) cytokines. WT responses were dominated by Th1 cytokines that were dramatically reduced in both DKO and TKO CD4 T cells (~5-fold) and CD8 T cells (~3-fold) (Fig. 7D). Th17 cytokines were highest in DKO CD4, and especially CD8 T cells, with markedly reduced levels detected in TKO cells that were, however, still ~ 4 times higher than in WT cells (Fig. 7E). In contrast, Th2 cytokines were highest in TKO T cells (Fig. 7F). This shift from Th17- to Th2-dominant responses mirrors the pattern seen in Fig. 3 analyzing DKO and TKO CD4 T cells responding against IAV in WT mice. Finally, CD8 T cells in DKO and TKO mice displayed ~ 6-fold reductions in Granzyme B and perforin compared to WT cells (Fig. 7G and H), consistent with low cytotoxic gene expression reported in DKO CD8 T cells in other studies.^46^ These results reveal striking numerical and functional differences in CD4 and CD8 T cell responses underling similar strengths of heterosubtypic immunity in WT, DKO, and TKO mice.

### Distinct lung inflammatory signatures in protected TKO versus DKO and WT mice

**Fig. 8.**
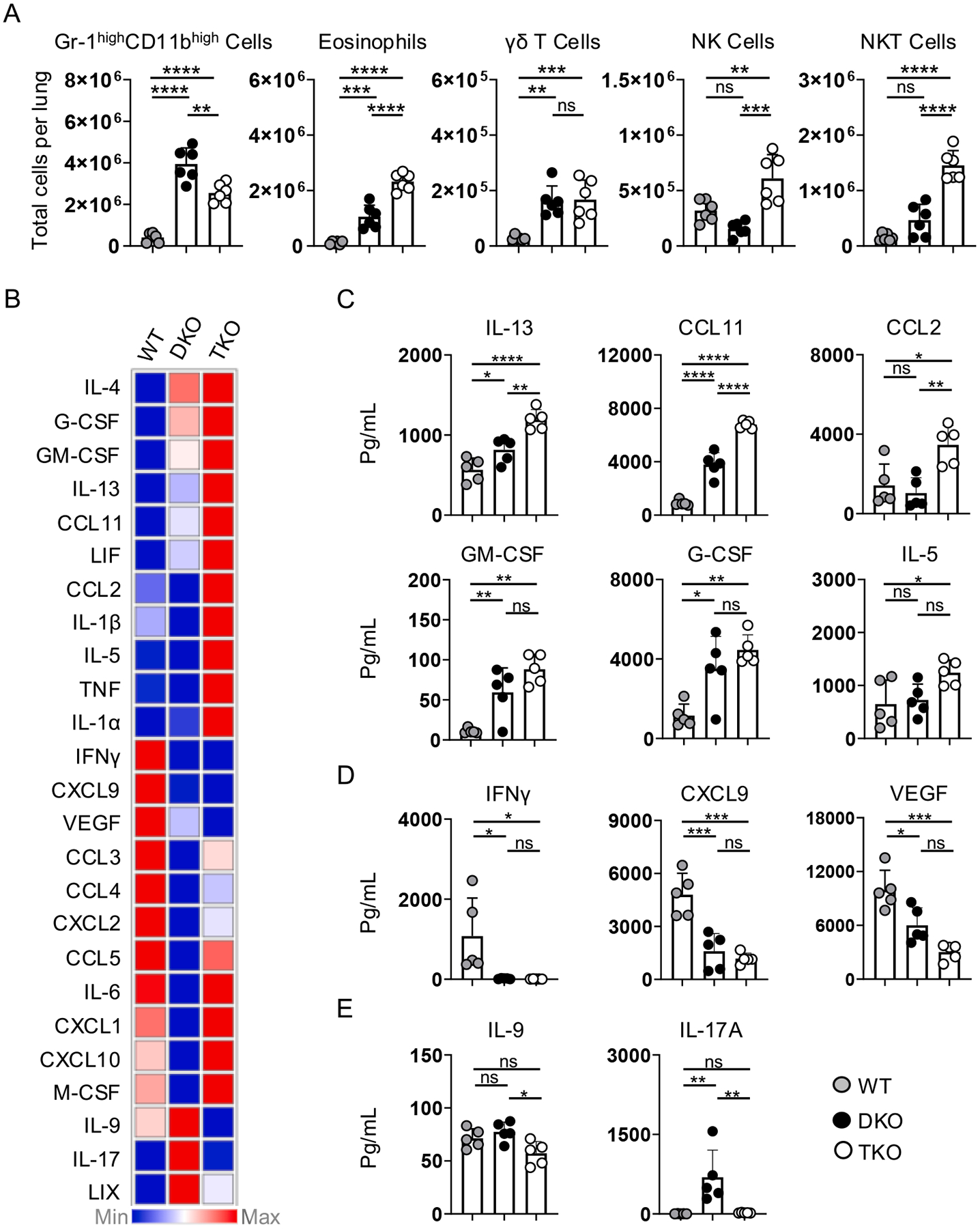
Heterosubtypic protection correlates with enhanced Th2-linked inflammatory patterns in TKO mice. WT, DKO, and TKO were primed with PR8 and challenged with A/Philippines as in Fig. 7. (A) At 4 dpi with A/Philippines, numbers of stated innate immune cell subsets were enumerated from 6 mice/group pooled from 2 experiments. (B) Heatmap depicting relative values for stated inflammatory factors determined by Luminex analysis of lung homogenates at 4 dpi. Protein concentrations detected by Luminex from lung homogenates from 5 mice/group correlating predominantly with (C) Th2, (D) Th1 or (E) Th17 inflammation; 1 of 2 experiments.

Finally, we assessed the innate immune response in the lungs during heterosubtypic infection in PR8-primed WT, DKO, and TKO mice 4 dpi with A/Philippines (Fig. 8A; see Supplemental Fig. 6 for gating strategies). Numbers of MHC-II^−^Gr-1^high^CD11b^high^ cells, that are highly enriched for neutrophils in the lungs of IAV-infected mice (McKinstry and Strutt, unpublished observations) but that could also contain Ly6C^high^ monocytes, were ~ 10-fold higher in DKO and TKO vs WT lungs. Eosinophils were also ~ 10-fold higher in DKO vs WT mice, and ~ 20-fold higher in TKO vs WT mice. Numbers of γδ T cells were ~ 5-fold higher in DKO and TKO versus WT mice, with more NK and NKT cells seen in TKO compared to WT and DKO mice. This analysis reveals striking differences in the involvement of major innate immune cell subsets between these mouse strains during successful viral clearance.

To further interrogate inflammatory environments, we harvested lung homogenates and determined protein levels of over 30 cytokines and chemokines. Several inflammatory factors were detected at similar levels across the strains, but we found significant differences in expression of 14 analytes between at least 2 groups, revealing dramatically distinct patterns (Fig. 8B and Supplemental Fig. 7). Those detected at the highest levels in TKO lungs, with significant differences compared to DKO mice, were IL-13, CCL11 (Eotaxin), and CCL2 (MCP-1), with a trend for higher GM-CSF, G-CSF, and IL-5 (Fig. 8C), all of which have strong ties to Th2-linked inflammation. Those most upregulated in WT lungs, IFNγ, CXCL9, and VEGF, are linked with Th1-linked responses (Fig. 8D), while those highest in DKO lungs, IL-9, and IL-17A, are associated with Th17-linked inflammation (Fig. 8E). Together, this analysis reveals a surprisingly broad spectrum of inflammatory responses, linked to the predominant Th-character of the antiviral memory T cell response, that correlate with effective heterosubtypic IAV clearance in mice with dominant Th1, Th2, or Th17 phenotypes.

## Discussion

Despite considerable evidence that CD4 T cells are key contributors to protection against IAV, discrete mechanisms by which they act are not clear. Th1 criteria define most virus-specific cells in mice and humans, which has led to a strong focus on Th1 hallmarks as correlates of protection. However, although Th1 identity is virtually abolished in IAV-primed DKO CD4 T cells, their protective capacity is similar to Th1-dominant WT cells.^26^ Given that the DKO cells not only lose Th1 imprints but develop strong Th17 identity, we hypothesized that transcriptional regulation directing maximal Th17 polarization would be required for their protective impacts. We show here, though, that CD4 T cells unable to support canonical Th1 or Th17 programming are undiminished in antiviral capacity compared to bona fide Th17 responders. Furthermore, we find that IAV-priming generates strong humoral immunity against homotypic reinfection, and T cell-dependent heterosubtypic immunity in TKO mice. These results reveal a remarkably broad continuum of functional T cell activation states, across and beyond the Th1 – Th17 spectrum, that can provide similar levels of protection against IAV.

We show that the relative capacity to produce Th1, Th17, or Th2 cytokines, as well as cytotoxic granules, cannot alone predict the efficacy of T cell responses targeting IAV. This is compatible with the concept that specific T cell attributes may gain or lose importance in settings where Th1- or Th17-inflammatory factors, or neither, dominates, leading to distinct modes of CD4 T cell immunity. The gene set upregulated by TKO vs DKO effectors primed under Th17 conditions may be helpful in further exploring this possibility by identifying targets involved in supporting novel, Th1/Th17-independent protective mechanisms. On the other hand, even marginal production of Th1 and/or Th17-associated factors may be sufficient to allow viral clearance by TKO cells through mechanisms that are shared with Th1 or Th17 responders. It will thus be important to delineate transcriptional control of the residual Th1/Th17 functionality during TKO responses. For example, STAT4 activation can promote some T-bet and Eomes-independent Th1 attributes during IAV infection,^26,47^ and Rorα can promote some Th17 attributes in the absence of Rorγt.^48^ Rorα induction requires receipt of TGFβ and IL-6 by CD4 T cells *in vitro*,^48^ signals we show are needed for Th17 cytokine production by TKO cells during IAV infection, making it an attractive target. However, other transcription factors, including c-Maf,^33^ which is upregulated in TKO vs DKO cells in IAV-infected mice, are also linked with Rorγt-independent IL-17 production. Interestingly, c-Maf^49^ and Rorα^50^ are also implicated in supporting Th2 functionality: we speculate that, in the absence of Rorγt, these regulators could support the dual IL-17^+^/IL-4^+^ subset that is enriched in TKO vs DKO effectors primed by IAV. The TKO platform described here may thus prove useful in exploring how antiviral T cells lacking strong Th1 and Th17 imprints can be harnessed through vaccination. Such responses may prove beneficial by avoiding pitfalls associated with strong production of Th1^10–15^ and Th17^51–52^ cytokines that can promote excessive immunopathology during IAV infection.

TKO cells producing Th17 (and Th2) cytokines were largely restricted to the lungs of IAV-infected mice, matching patterns in our previous studies assessing DKO Th17 responses.^26^ These observations are in line with the concept that T cells continue to be impacted by inflammatory signals well after their initial priming, most likely during cognate interactions with antigen presenting cells, that can further direct their phenotypic, transcriptional, and functional profiles.^53^ That Th17 and Th2 cytokine production by TKO and DKO OT-II cells responding in IAV-infected lungs was higher than seen in Th17 or Th2 cultures suggests that such interactions may provide signals to maximize effector functions that are not received *in vitro*. As antigen-dependent interactions are also critical in promoting memory fitness of IAV-primed CD4 T cells,^54^ improved understanding of this timeframe of the response is crucial in the development of IAV vaccines able to better promote T cell memory.

Resistance to bacterial superinfection has been shown to require the rapid induction of innate Th17-linked inflammation, which is normally restricted by Th1 imprints in the lungs during viral infection.^17,40–42^ As Th17 inflammatory signatures are blunted in IAV-infected TKO vs DKO mice, we hypothesized that TKO mice would be highly susceptible to *Spn* superinfection. However, IAV-infected TKO mice rapidly cleared *Spn* and displayed strong resistance to superinfection-induced morbidity matching outcomes seen in T-bet KO and DKO mice. Further studies are required to determine if residual Th17-linked factors in TKO mice are sufficient to support superinfection resistance, or if other mechanisms contribute. For example, TKO CD4 T cells produced higher levels of IL-10 and IL-4 than DKO CD4 T cells, and both of these cytokines have been shown to also contribute to superinfection resistance^55,56^.

Defining innate immune signatures associated with positive versus negative outcomes of IAV infection is an important goal in more accurately predicting disease severity and improving the management of clinical cases. Our analysis here, rather than identifying elements of a conserved hierarchy of cytokine and chemokine production and innate immune cell involvement spanning protection across WT, DKO, and TKO mice, indicates that multiple, complex inflammatory patterns can be associated with similar efficiency of heterosubtypic IAV clearance. Leveraging strategies to identify personalized biomarkers, rather than broadly applicable innate response phenotypes, may thus be needed to capture the predictive potential of this domain of measurements within diverse populations. Our results are also compatible with the concept that different subsets of innate immune cells may gain or lose importance in determining outcomes depending on the relative Th1/Th17 balance of the antiviral immune response.

In summary, we find that ‘master regulator’ transcription factors controlling the polarization of both Th/Tc1 and Th/Tc17 lineages are dispensable for clearance of primary IAV infection and the generation of robust T cell-dependent protection against reinfection with heterosubtypic IAV strains. Our results thus reinforce the need for a more refined mechanistic understanding of effective antiviral T cell responses, and how they integrate with innate inflammatory constituents to promote protective outcomes. We suggest that analysis unbiased by Th-subset paradigms can provide an important approach towards this goal and the development of IAV vaccines able to fully harness antiviral T cell immunity.

## Supplementary Material

1

2

## Appendix A. Supplementary data

Supplementary data to this article can be found online at https://doi.org/10.1016/j.mucimm.2025.08.005.
