# Supplementary material for: T-cell immunity against influenza virus does not require Th1 or Th17 master regulator transcription factors
Source: Mucosal Immunol. Author manuscript; Available in PMC 2025 Dec 26. (PMC12741987; doi:10.1016/j.mucimm.2025.08.005)
Supplement: 1 [file NIHMS2129403-supplement-1.pdf]

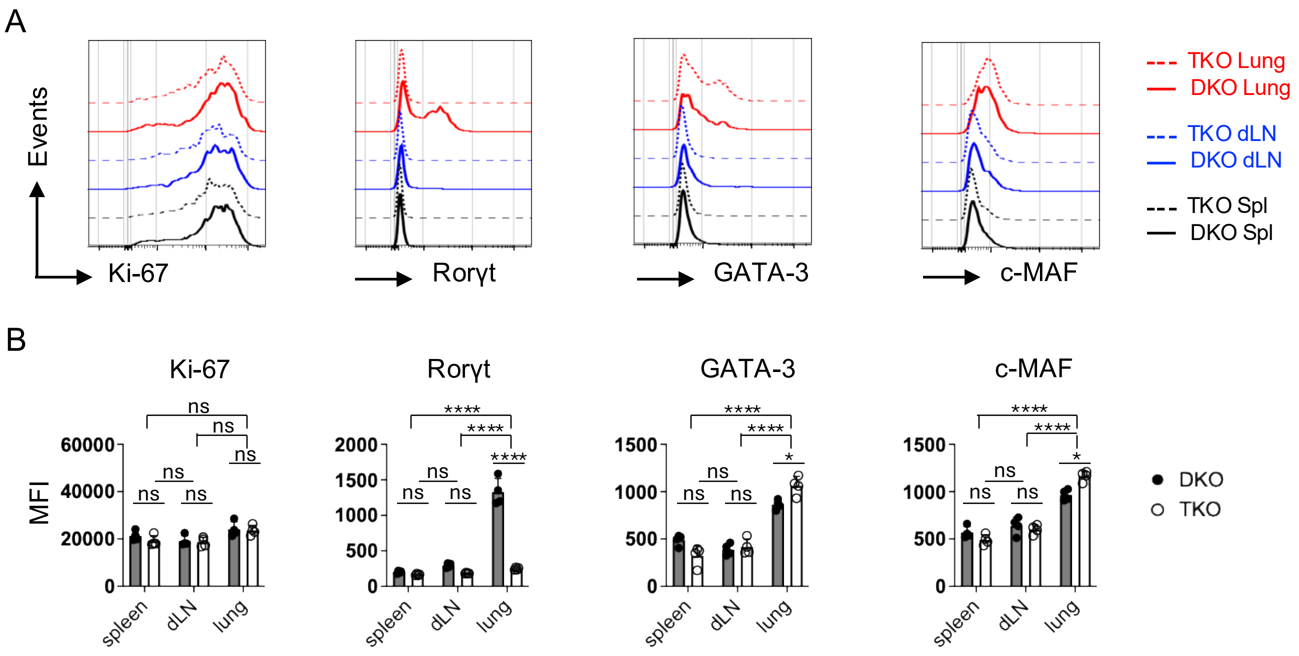


**Supplemental Figure 1:** 5x10$^5$ naive CD45.2$^+$ DKO or TKO OT-II cells were transferred to unprimed CD45.1$^+$ WT hosts that were then challenged with 0.25 LD$_{50}$ PR8-OVA$_{II}$. **(A)** Representative staining for stated factors by TKO (dashed) or DKO (solid) donor cells responding in the lung (red), dLN (blue), and spleen (black) at 7 dpi. **(B)** MFI analysis from 4 mice/group for DKO (black) and TKO (white) cells responding in stated organs. Significance determined by 2way ANOVA with Tukey's multiple comparisons test.

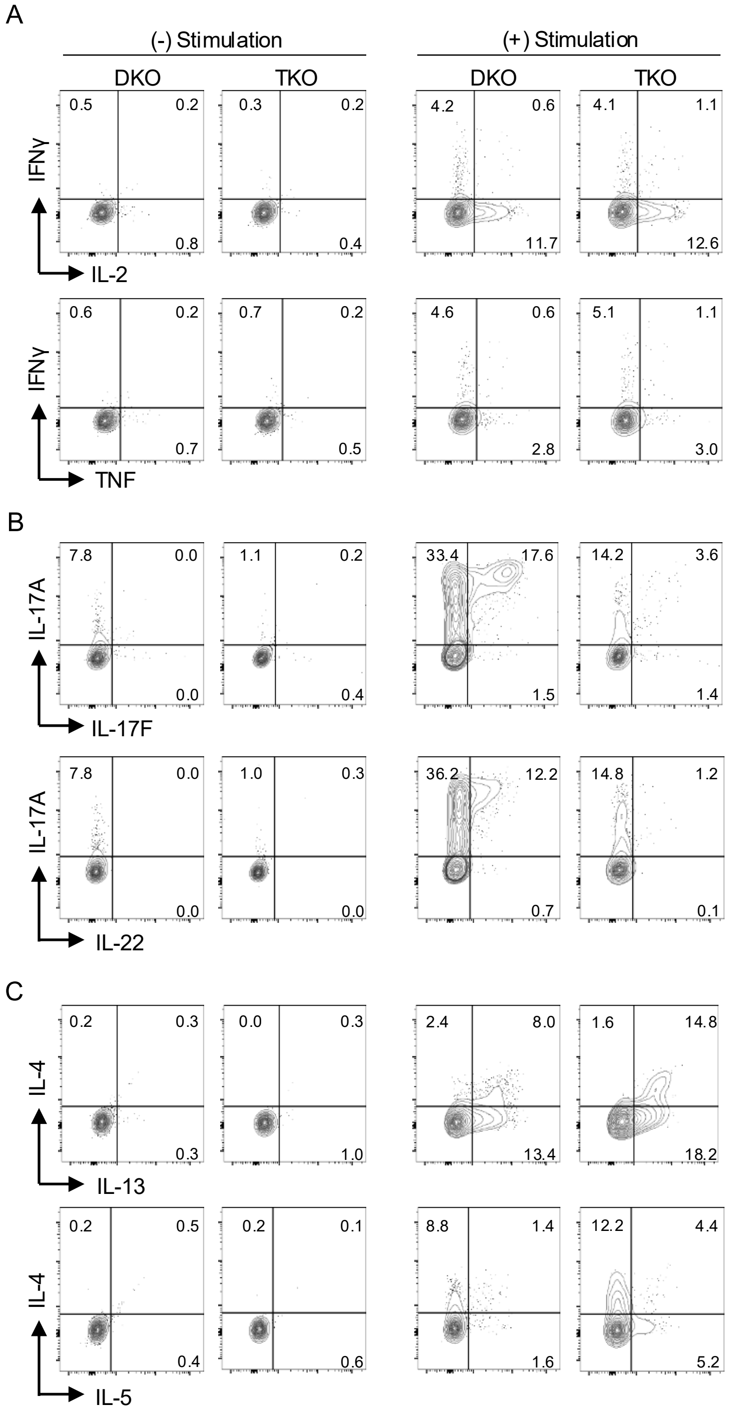


**Supplemental Figure 2:** 5x10[5] naive CD45.2[+] DKO or TKO OT-II cells were transferred to unprimed CD45.1[+] WT hosts that were then challenged with 0.25 $LD_{50}$ PR8-$OVA_{II}$. Representative intracellular cytokine staining of DKO and TKO cells responding in the lungs at 7 dpi without (left panels) or after restimulation (right panels) for **(A)** IFNγ, TNF, and IL-2; **(B)** IL-17A, IL-17F, and IL-22; and **(C)** IL-4, IL-5, IL-13.

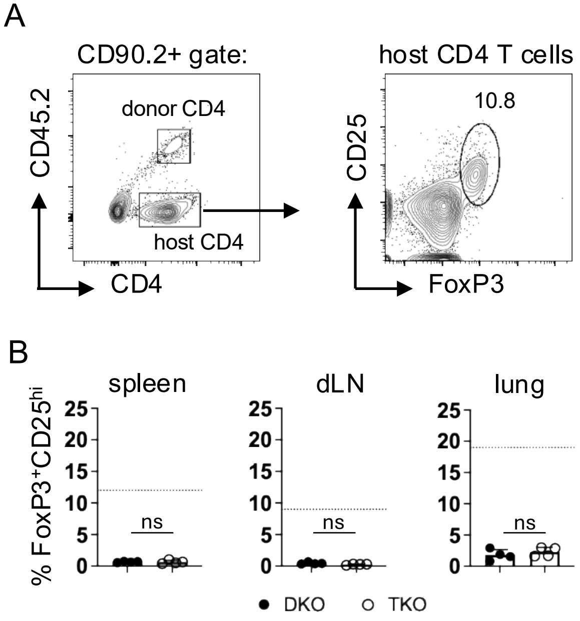


**Supplemental Figure 3:** 5x10$^{5}$ naive CD45.2$^{+}$ DKO or TKO OT-II cells were transferred to unprimed CD45.1$^{+}$ WT hosts that were then challenged with 0.25 LD$_{50}$ PR8-OVA$_{II}$. At 7 dpi, the frequency of host and donor Tregs (FoxP3$^{+}$CD25$^{high}$) was determined using **(A)** Treg staining of host CD4 T cells to set gating parameters, with representative staining shown. **(B)** The frequencies of donor DKO and TKO Tregs detected in stated organs of separate host mice; 4 mice/group.

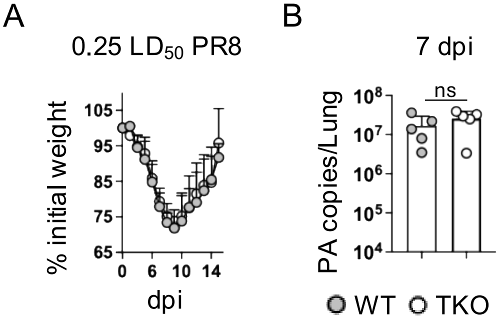


**Supplemental Figure 4**: Unprimed WT or TKO mice were challenged with 0.25 $LD_{50}$ PR8. **(A)** Weight loss and survival from groups of 6 mice. **(B)** Lung viral titers analysis at 7 dpi; 5 mice/group.

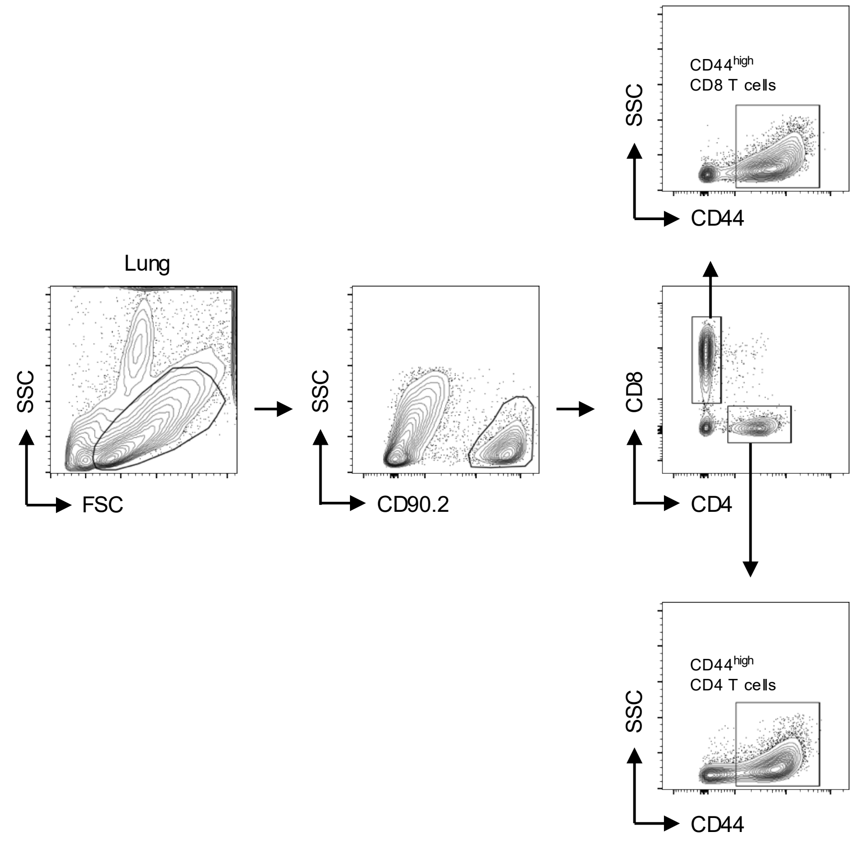


**Supplemental Figure 5:** Representative gating used to identify CD44$^{high}$ CD4 and CD8 T cells in the lungs of mice primed with PR8 at 4 dpi with A/Philippines. Representative staining depicting detection of tetramer$^{+}$ CD4 and CD8 T cells within the CD44$^{high}$ gates is shown in Figure 7 A and B.

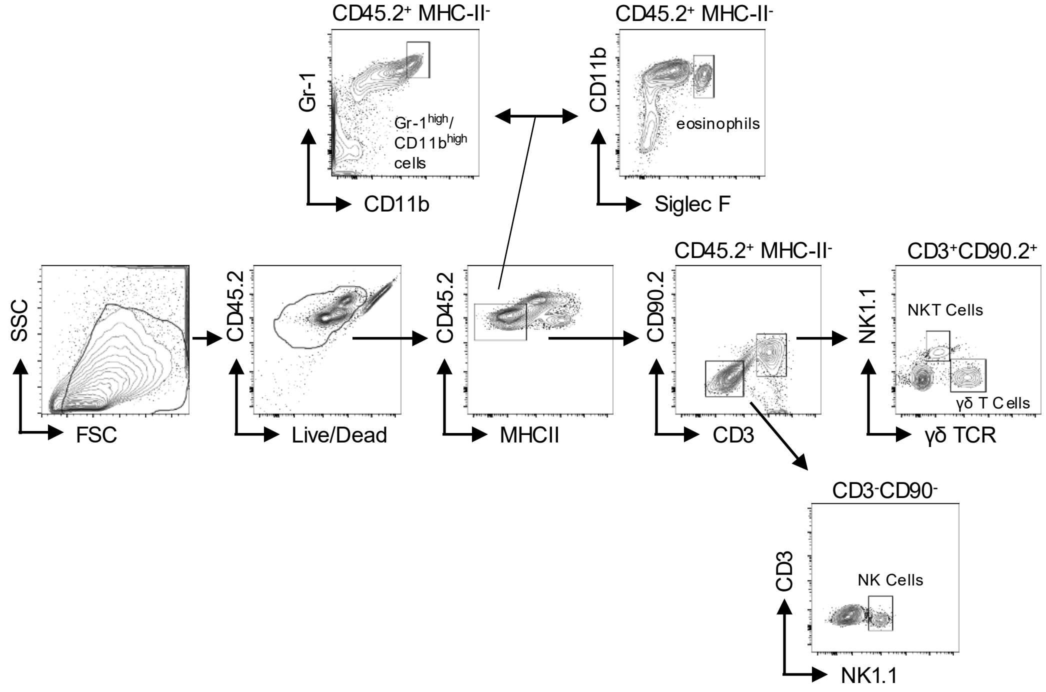


**Supplemental Figure 6:** Representative FACS staining depicting the gating strategy used to identify innate immune subsets summarized in Figure 8 in lungs of mice primed with PR8 at 4 dpi with A/Philippines.

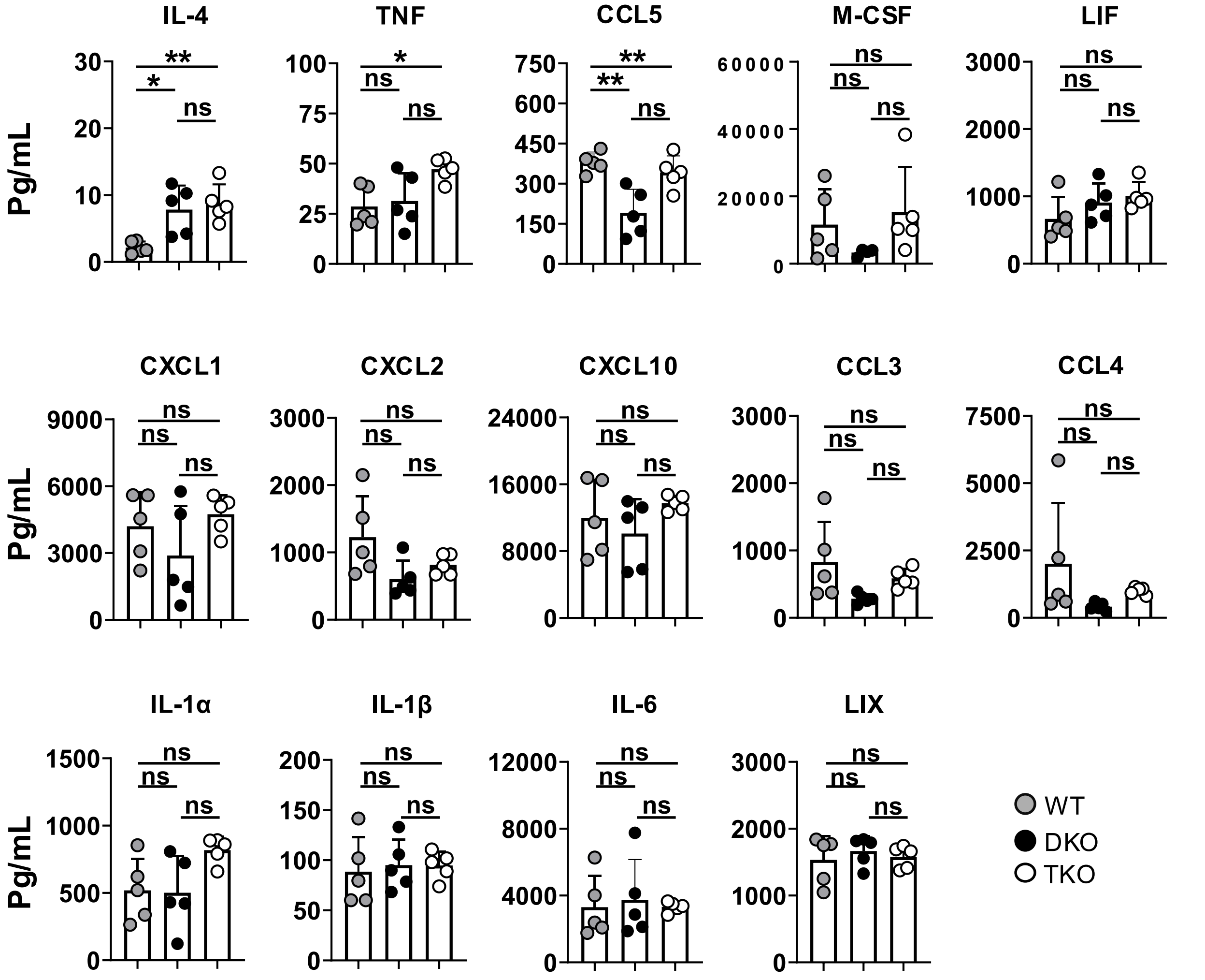


**Supplemental Figure 7:** Values for stated analytes detected from lung homogenates harvested from PR8-primed mice 4 dpi with A/Philippines. 5 individual mice/group; 1 of 2 experiments. Note: IL-2, IL-3, IL-7, IL-12p40 AND IL-12 p70 were not detected above background levels.
